# Tracing temperature in a nanometer size region in a picosecond time period

**DOI:** 10.1038/srep13363

**Published:** 2015-08-21

**Authors:** Kaoru Nakajima, Takumi Kitayama, Hiroaki Hayashi, Makoto Matsuda, Masao Sataka, Masahiko Tsujimoto, Marcel Toulemonde, Serge Bouffard, Kenji Kimura

**Affiliations:** 1 Department of Micro Engineering, Kyoto University, Kyoto 615-8540, Japan; 2 Nuclear Science Research Institute, Japan Atomic Energy Agency, Tokai, Naka, Ibaraki 319-1195, Japan; 3 Institute for Integrated Cell-Material Sciences, Kyoto University, Kyoto 606-8501, Japan; 4 CIMAP-GANIL (CEA-CNRS-ENSICAEN-Université de Caen Basse Normandie), Bd. H. Becquerel, 14070 Caen, France

## Abstract

Irradiation of materials with either swift heavy ions or slow highly charged ions leads to ultrafast heating on a timescale of several picosecond in a region of several nanometer. This ultrafast local heating result in formation of nanostructures, which provide a number of potential applications in nanotechnologies. These nanostructures are believed to be formed when the local temperature rises beyond the melting or boiling point of the material. Conventional techniques, however, are not applicable to measure temperature in such a localized region in a short time period. Here, we propose a novel method for tracing temperature in a nanometer region in a picosecond time period by utilizing desorption of gold nanoparticles around the ion impact position. The feasibility is examined by comparing with the temperature evolution predicted by a theoretical model.

The interaction of energetic ions with materials is the basis of a wide range of applications, such as surface analysis, surface modification, ion implantation and so on. Recently, nanostructures produced by single ion impact is attracting a wide attention because of its potential applications. When a swift heavy ion (SHI) penetrates a solid the ion excites solid electrons. The energy of the excited electrons is then transferred to the lattice via electron-phonon coupling and provides ultrafast local heating along the ion path. Eventually, a cylindrical damage region of diameter several nm, a so-called ion track, may be created when the electronic energy loss is larger than a material dependent threshold value^1^. Such ion tracks are used for DNA sequencing^2^, templates for the synthesis of micro- and nanowires^3^, and waveguide-mode biosensors^4^ and so on. The formation mechanism of ion track is explained by a so-called inelastic thermal spike (i-TS) model^1^. In the i-TS model, the evolution of the temperature distribution around the ion path is described by classical heat equations for the electronic and atomic subsystems. It is generally assumed that the ion track is formed when the atomic temperature rises beyond the melting point of the material^3^. Because such heating occurs in a highly localized region of nanometer size on a time scale of ~10 ps, it is very difficult to confirm the assumption by tracing the temperature during the track formation.

Similar ultrafast heating can be also realized by the irradiation of pulsed lasers. When a solid target is illuminated with a pulsed laser, the solid electrons are excited and the deposited energy is transferred to the phonon system on a picosecond time scale. This phenomenon is the basis of laser ablation which has been widely used for the deposition of a wide range of materials. The laser ablation is often described by the so-called two temperature model^5^,^6^, which is basically the same model as the i-TS model. Recently it was demonstrated that ultrafast heating in localized region can be realized by combing the pulsed laser and local plasmon resonance^7^. When gold nanoparticles are illuminated by a pulsed laser at their plasmonic resonance, the laser power is deposited into the electronic subsystem of the nanoparticles through the plasmon resonance. The deposited energy is then transferred to the atomic subsystem via electron-phonon coupling. This is called pulsed laser plasmon-assisted photothermal heating^6^ and is a promising heat source of nanometer size in ultra-fast time frames. Theoretical studies showed that the temperature of nanoparticles rises ~1000 K in a nanosecond time period^8^ although the measurement of the actual temperature is very difficult.

More recently, it was found that individual slow highly charged ions (HCI) produce surface modifications (either hillock, pits or craters) on a nanometer scale when the potential energy carried by HCI is larger than a material dependent threshold value^9^,^10^,^11^,^12^,^13^,^14^. These modifications result from the large potential energy (*e.g.* ~16 keV for Xe^30+^) carried by slow HCI. The potential energy is first deposited to the surface electrons in a nanometer region and then transferred to the atomic system. This leads to ultrafast local heating around the ion impact position. The observed potential energy threshold for hillock formation was well reproduced by the i-TS calculation assuming that the hillock is formed when the temperature rises beyond the melting point^15^.

All these phenomena are similar in the sense that the initial energy deposition to the electronic subsystem results in ultrafast local heating of the atomic subsystem. Although theoretical studies predict the evolution of temperature distribution there has been no direct temperature measurement of such ultrafast local heating. Based on the molecular dynamics (MD) simulations that determine the surface desorption energy of gold nanoparticles^16^, we propose a novel method to trace temperature in highly localized region on a ultrafast time scale. Thin films deposited with gold nanoparticles are irradiated with swift heavy ions and the desorption of nanoparticles around the ion impact position is observed using transmission electron microscopy (TEM). The feasibility of this method will be examined by comparing the observed radius in which the nanoparticles are expelled with the i-TS model calculations.

## Results

### Desorption of gold nanoparticles

**Figure 1:**
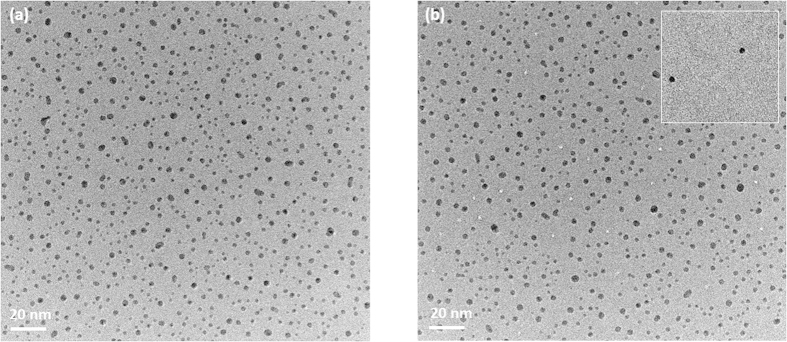
TEM bright field images of gold-deposited a-SiO_2_ films. (**a**) The as-deposited sample, (**b**) the samples after irradiation with 420 MeV Au ions on the rear surface are shown. The ion tracks are seen as bright spots. The gold nanoparticles disappeared from the vicinity of the ion track. The inset of Fig. 1(b) shows a TEM image of a collector foil.

**Figure 2:**
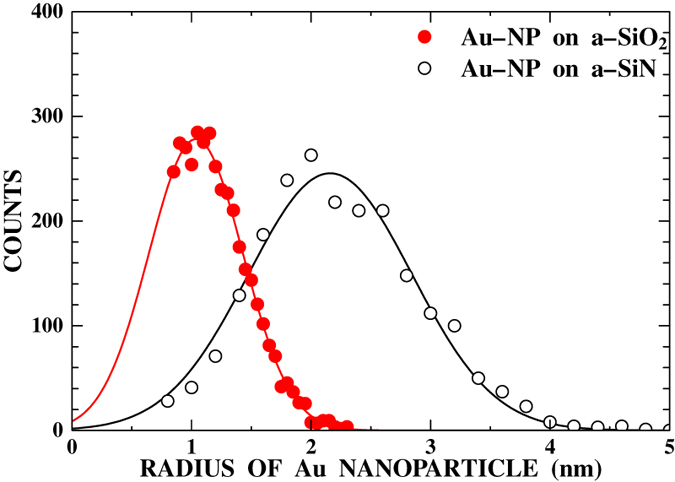
Size distributions of gold nanoparticles deposited on a-SiO_2_ (closed circles) and a-SiN (open circles). The lines show the results of Gaussian fitting.

Figure 1(a) shows an example of TEM bright field images of a gold-deposited amorphous SiO_2_ (a-SiO_2_) film (thickness 20 nm) observed before irradiation. There are many gold nanoparticles formed by the gold vapor deposition. The areal density, *N*, of these nanoparticles was measured to be 1.9 × 10^12^  particles/cm^2^. The size distribution of these nanoparticles was derived from the observed TEM images and shown by closed circles in Fig. 2. The distribution shows a Gaussian-like well-defined peak with a peak radius of 1.0 nm and a width of 0.9 nm. A similar size distribution with a peak radius of 2.2 nm and a width of 1.6 nm was also observed for the gold nanoparticles deposited on amorphous SiN (a-SiN) films (thickness 30 nm) as shown by open circles in Fig. 2. The size difference between a-SiO_2_ and a-SiN is attributed to the smaller diffusivity of gold adatoms on a-SiO_2_ surfaces compared to a-SiN.

**Figure 3:**
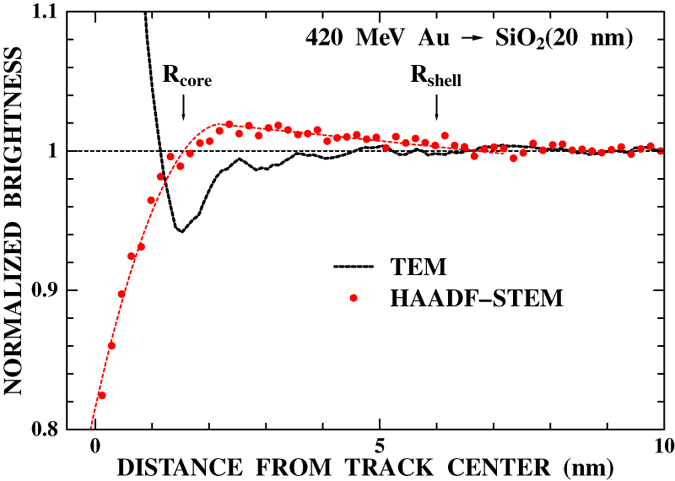
Radial density profile of the ion track derived from the observed HAADF-STEM images of a-SiO_2_ films irradiated with 420 MeV Au ions (solid circles). The intensity profile derived from the observed TEM images is also shown for comparison (dashed line).

Figures 1(b) shows an example of TEM bright field images of the gold-deposited a-SiO_2_ film observed after irradiation with 420 MeV Au ions. The irradiation was performed on the rear surface, *i.e.* from the opposite side of the gold deposition (will be referred to as “rear surface irradiation”). Ion tracks are clearly seen as bright spots with a diameter of about 2 nm. The ion tracks were also observed using high-angle annular dark field scanning transmission electron microscopy (HAADF-STEM). The profiles of the observed track images are shown for both TEM and HAADF-STEM in Fig. 3. The TEM profile has an oscillatory structure caused by Fresnel diffraction, indicating the difficulty of deducing quantitative information from TEM images. On the contrary, the HAADF-STEM profile can be directly linked to the density profile. The observed HAADF-STEM profile shows a core-shell structure, namely a low density core (radius 1.6 ± 0.3 nm) surrounded by a high density shell (outer radius 6 ± 1 nm). This is in good agreement with the observation using small angle x-ray scattering^17^. According to the two thresholds model^17^,^18^ (see also Supplementary information), the present result suggests that the energy per atom surpasses the melting energy (0.38 eV/atom^17^) at *r* < 6 ± 1 nm and boiling energy (1.7 eV/atom^17^) at *r* < 1.6 ± 0.3 nm.

**Figure 4:**
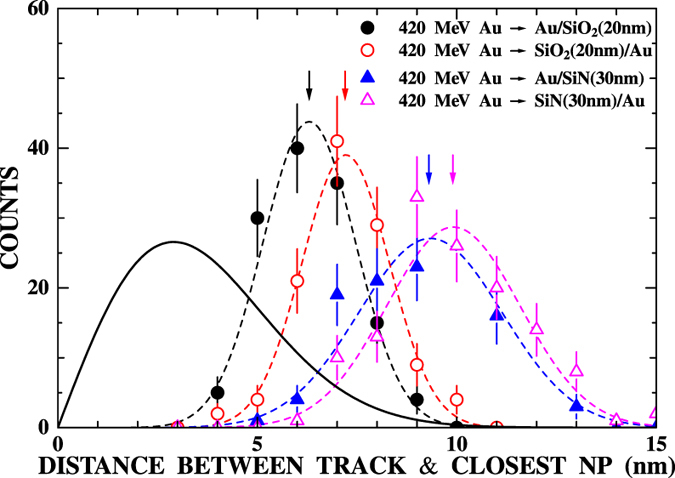
Distributions of the distance between the ion track and the closest gold nanoparticle. The results for the irradiation of a-SiO_2_ with 420 MeV Au ions on the front surface (solid circles) and on the rear surface (open circles) are shown. The results of a-SiN are also shown by solid triangles (front surface irradiation) and open triangles (rear surface irradiation). The arrows show the average distances. The dashed lines show the results of Gaussian fitting. The solid line shows the calculated distribution if nanoparticles are not desorbed by the ion impact (see text).

Looking at the vicinity of the ion track closely (Fig. 1(b)), the gold nanoparticles seem to disappear from the surrounding area of the ion tracks. Such disappearance of the gold nanoparticles was also observed for the front surface irradiation. The distance between the ion track and the closest gold nanoparticle was measured for each ion track. The observed closest distance, *R*_c_, represents the radius of the region where nanoparticles disappeared. The distributions of the measured closest distances for the samples irradiated on the front surface (solid circles) and the rear surface (open circles) are shown in Fig. 4. The average of the closest distance, <*R*_c_>, is 6.3 ± 0.6 and 7.2 ± 0.7 nm for the front and rear surface irradiations, respectively. This suggests that 2.4 and 3.1 nanoparticles, on average, are removed by single Au ion impact. In order to confirm this, the distribution of closest distance was calculated assuming that there was no removal of nanoparticles around the ion tracks. The probability that the closest nanoparticle is found in a region [*R*_c_, *R*_c_ + *dR*_c_] is given by
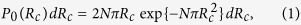
if the gold nanoparticles are not desorbed by the ion irradiation. The distribution was calculated with the observed *N* (1.9 × 10^12^  particles/cm^2^) and shown by a solid line in Fig. 4. The calculated distribution has a peak at a distance of ~3 nm, which is much smaller than the observed ones (6.3 and 7.2 nm). This clearly indicates that nanoparticles are really removed from the vicinity of the ion impact position.

The removed nanoparticles were collected by a thin foil placed in front of the gold-deposited surface of the sample. This collector foil was also observed using TEM. An example of the TEM images is shown in the inset of Fig. 1(b). Very precisely circular gold particles with a radius close to those of the deposited gold nanoparticles were observed, indicating that gold nanoparticles were desorbed as liquid droplets without fragmentation from the surface of a-SiO_2_ by the ion irradiation.

Similar desorption of gold nanoparticles upon ion impact was also observed for the gold-deposited a-SiN films. The measured distributions of the distance between the ion track and the closest nanoparticle are shown by solid and open triangles for front and rear surface irradiations, respectively, in Fig. 4. The average closest distances were derived to be 9.3 ± 0.9 and 9.9 ± 1.0 nm for front and rear surface irradiations, respectively. It is noteworthy that the nanoparticle cleared region is larger in the case of the rear surface irradiation compared to the front surface irradiation for both a-SiO_2_ and a-SiN. This will be discussed later in this paper.

If the density of the nanoparticle is infinitely large, the measured closest distance *R*_c_ between the track center and the surrounding nanoparticles is equal to the radius of the nanoparticle cleared region, *R*. The actual density is, however, finite. As a result, the measured closest distance is larger than *R*. The relation between <*R*_c_> and *R* can be derived using a Poisson law. The probability that the closest nanoparticle is found in a region [*r, r* + *dr*] is given by
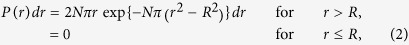
where *r* is the distance from the track center. The average of the closest distance is calculated by,
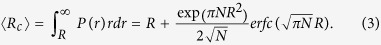
Using Eq. (3), *R* was estimated from the observed <*R*_c_> and *N*. The results are summarized in Table 1.

Anders *et al.* studied the desorption mechanism of nanoparticles using MD simulations^16^. They simulated the motion of hemispherical gold nanoparticles (radius 3.6 nm) placed on a surface when the gold atoms are energized. The simulation was performed for various initial kinetic energies given to the gold atoms ranging from 0.13 to 1.03 eV/atom. They found that the nanoparticles are desorbed within a short time period of several ps without fragmentation when the initial kinetic energy exceeds a threshold energy, *E*_d_ = 0.4 eV/atom. Their detailed investigation showed that the desorption is triggered by melting of nanoparticles, which leads to expansion of the nanoparticles and results in the desorption. This is in accordance with the present observation that the gold nanoparticles were desorbed as liquid droplets without fragmentation. In view of their result, present observation indicates that the energy deposited on the atoms exceeds 0.4 eV/atom in the region closer than *R* around each ion impact position. It is noteworthy that the observed nanoparticle cleared radii for a-SiO_2_ (5.1 ± 0.6 and 6.0 ± 0.7 nm, see Table 1) are very close to the shell radius of the observed track (6 ± 1 nm). This indicates that the threshold energy for track shell formation is close to the threshold energy for desorption (*E*_d_ = 0.4 eV/atom). Actually, the threshold energy for track shell formation was estimated to be 0.38 eV/atom for a-SiO_2_^17^,^19^. This suggests that the proposed method of the temperature tracing works well.

### Comparison with the result of i-TS calculation

The evolution of the temperature distribution around the impact position was calculated using the i-TS model. In the i-TS model, the heat diffusion in time *t* and space *r* (radial distance from the ion path) is described by the following differential equations^1^,
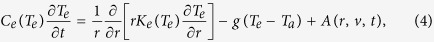

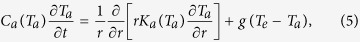
where *T*_*e,a*_*, C*_*e,a*_ and *K*_*e,a*_ are the respective temperature, specific heat and thermal conductivity of the electronic and atomic subsystems, *g* is the electron-phonon coupling parameter, *A*(*r, v, t*) is energy input into the electronic subsystem from the electronic energy loss, and *v* is the ion velocity. The energy lost by the projectile ion is sheared between electrons and then gradually transferred to atoms by the electron-phonon interaction. The parameter *g* is related to the electron-phonon mean free path *λ*. For a-SiO_2_, this parameter is well established, *i.e. λ* = 3 nm^17^,^19^. With this mean free path, the i-TS model reproduces observed track radii in a-SiO_2_ very well^17^,^19^.

Unlike with a-SiO_2_, the electron-phonon coupling parameter for a-SiN is not well known. For crystalline Si_3_N_4_, *λ* can be estimated to be 4.3 nm from the empirical relation between *λ* and band gap energy (5.3 eV for Si_3_N_4_)^20^. Considering the fact that *λ* for amorphous materials is significantly lower than that for the same material in its crystalline phase, we use *λ* = 3 nm for a-SiN. This was chosen so that the observed track radii in a-SiN^21^,^22^ can be reproduced (see Supplementary information).

**Figure 5:**
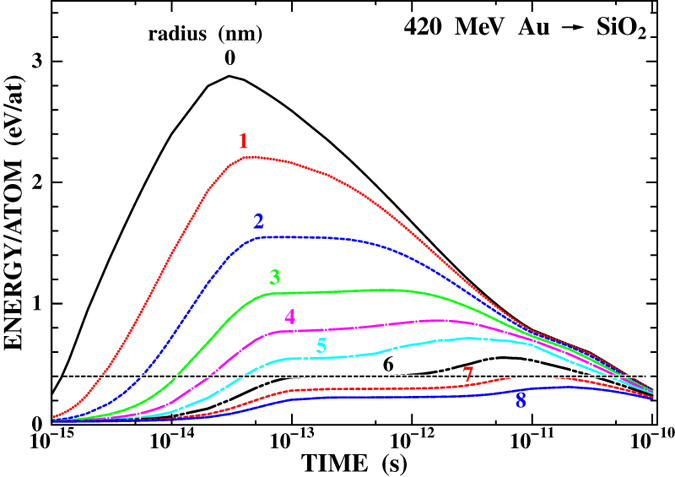
Result of the calculation of the i-TS model for a-SiO_2_ irradiated with 420 MeV Au ions. The energy deposited on the target atoms is shown as a function of time at different radial distances from the projectile trajectory. The horizontal line indicates the threshold energy for desorption of gold nanoparticles.

With the above mentioned parameters, the evolution of the temperature distribution around the ion impact position was calculated using the i-TS model. Figure 5 shows the result of a-SiO_2_. The horizontal line indicates the threshold energy for desorption (*E*_d_ = 0.4 eV/atom^16^). The energy deposited on the atoms exceeds the threshold energy when the distance from the impact position *r* is smaller than 7 nm. This local heating continues for more than picosecond. The similar calculation was performed for the a-SiN irradiated with the 420 MeV Au ion. The obtained critical radius within which the deposited energy exceeds the threshold energy for desorption is 7.6 nm. These calculated critical radii are roughly in agreement with the observed nanoparticle cleared radii *R*, indicating that the i-TS model reproduces the temperature distribution during the track formation. It is also noteworthy that i-TS calculation also reproduces the observed track radius. From Fig. 5, it can be seen that the energy deposited on atoms exceeds the energy to boil (1.7 eV/atom for a-SiO_2_^17^) and the energy to melt (0.38 eV/atom^17^) at *r* smaller than 1.7 and 7.2 nm, respectively. These radii are in good agreement with the observed track core and shell radius, 1.6 ± 0.3 nm and 6 ± 1 nm, respectively, for a-SiO_2_.

**Figure 6:**
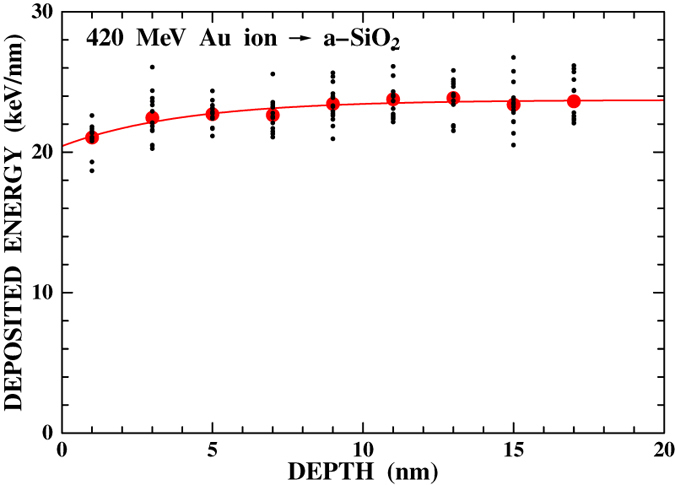
Evolution of the deposited energy along the ion path calculated using Monte Carlo simulation when 420 MeV Au ion travels through a-SiO_2_. Small dots show the results of each simulations and the closed circles show the mean value. The solid line shows the result of exponential curve fitting.

Finally the difference in the nanoparticle cleared radius between the front and rear surface irradiations is discussed. A possible origin of the observed difference is the effect of high-energy secondary electrons (so-called δ-rays) produced by the projectile ions. The δ-rays carry away the deposited energy and do not contribute to heating the place of production but do contribute in the deeper region. As a result, the deposited energy is smaller than the energy loss in the entrance region and increases with depth. The evolution of the deposited energy along the ion path was calculated using Monte Carlo simulations^23^. Figure 6 shows the result of the MC simulations for 420 MeV Au ions travelling through a-SiO_2_. The result of each simulation is shown by small dots and the averaged result is shown by solid circles. The result was fitted by an exponential function and is shown by a solid line. The deposited energy at the entrance surface is 20.4 keV/nm and increases with depth. Eventually, it reaches equilibrium at ~15 nm. The deposited energy at the exit surface of the 20-nm a-SiO_2_ film is 23.7 keV/nm, which is about 16% larger than that of the entrance surface. Using these deposited energies, the radii of the region where the energy per atom exceeds *E*_d_ were calculated to be 6.4 nm and 7.0 nm at the entrance and exit surfaces, respectively, for a-SiO_2_. These radii are slightly larger than the observed results, 5.1 ± 0.6 and 6.0 ± 0.7 nm (see Table 1), but the difference between the front and rear irradiation is well reproduced. For a-SiN, the calculated radii are 6.9 and 7.6 nm at the entrance and exit surfaces, respectively, showing good agreement with the observed ones, 7.5 ± 0.9 and 8.1 ± 1.0 nm (see Table 1). These results demonstrate that the desorption of gold nanoparticles can be used to measure the temperature of the localized area of nm size in a short time period. In passing we note that similar measurements of nanoparticle desorption from a-SiN films of different thicknesses upon impact of 0.72 and 1.1 MeV C_60_ ions were performed using different kinds of nanoparticles, platinum and gold nanoparticles with different sizes. In spite of very different experimental conditions, the observed nanoparticle cleared radii agree with the i-TS calculations. This supports that the present approach is a robust method.

In conclusion, gold nanoparticles were deposited on a-SiO_2_ and a-SiN films and irradiated with 420 MeV Au ions. The irradiated samples were observed using TEM and HAADF-STEM. The ion tracks produced by the ion irradiation are clearly seen and the gold nanoparticles are found to be cleared from the vicinity of each ion track. The radii of the nanoparticle cleared regions were derived to be 5.1 ± 0.6 and 6.0 ± 0.7 nm for front and rear irradiation of a-SiO_2_, respectively, and 7.5 ± 0.9 and 8.1 ± 1.0 nm for a-SiN. The radius of the region where the energy deposited on the atoms exceeds 0.4 eV/atom, which is the threshold energy for the desorption of gold nanoparticles predicted by the MC simulations, was calculated using the i-TS model. The calculated radii are in reasonable agreement with the observed nanoparticle cleared radii both for a-SiO_2_ and a-SiN. The observed difference in the nanoparticle cleared radius between the front and rear surface irradiations was attributed to the effect of δ–rays and quantitatively reproduced by the i-TS calculations in combination with MC simulations of δ–rays. The present result demonstrates that the desorption of gold nanoparticles can be utilized for the temperature measurement in a nanometer size region in a picosecond time period. More detailed temperature distribution may be deduced using nanoparticles of other materials with different melting points (see Supplementary information).

## Methods

### Preparation of samples

Self-supporting a-SiO_2_ (thickness 20 nm) and a-SiN (thickness 30 nm) films were purchased from Structure Probe, Inc. and Silson Ltd, respectively. The composition of the a-SiN film was determined to be Si_0.47_N_0.53_ using high-resolution Rutherford backscattering spectrometry^24^, which is slightly Si rich compared to the stoichiometric Si_3_N_4_. A small amount of gold was vapor deposited on one side of the films at room temperature under a vacuum of 10^−4^ Pa. The average thickness of the deposited gold is several tenth of nanometer.

### Irradiations and TEM measurements

The gold-deposited films were irradiated with 420 MeV Au ions at normal incidence to a fluence of ~5 × 10^10^ ions/cm^2^ at the tandem accelerator facility of the Japan Atomic Energy Agency (JAEA). A carbon foil (20 μg/cm^2^) was placed in front of the samples to acquire an equilibrium charge state. This carbon foil was also used as a collector foil, *i.e.* used to capture particles emitted from the target during irradiation. The irradiation was performed either on the gold-deposited side (“front surface irradiation”) or on the rear side (“rear surface irradiation”) at normal incidence. In the rear surface irradiation, a self-supporting a-SiO_2_ film was placed just behind the sample (facing to the gold-deposited surface) to capture gold nanoparticles emitted from the film during the irradiation. Both the irradiated samples and the collector foils were observed using TEM (JEOL JEM-2200FS) equipped with a field emission gun operating at 200 kV. The images were taken by GATAN Ultrascan 1000 CCD camera with a 2 k × 2 k pixel. For the observation of HAADF-STEM, the electron beam was converged to 0.5 nm in diameter and an annular dark detector covering over 120 mrad were used.

## Additional Information

**How to cite this article**: Nakajima, K. *et al.* Tracing temperature in a nanometer size region in a picosecond time period. *Sci. Rep.* **5**, 13363; doi: 10.1038/srep13363 (2015).

## Supplementary Material

Supplementary Information

## Figures and Tables

**Table 1:** Average of the observed distances between the ion track and the closest gold nanoparticle (<*R*_c_>), the radius of nanoparticle cleared region (*R*) derived from <*R*_c_> and the result of the i-TS calculation.

	a-SiO_2_		a-SiN	
	**front irradiation**	**rear irradiation**	**front irradiation**	**rear irradiation**
<*R*_c_> (nm)	6.3 ± 0.6	7.2 ± 0.7	9.3 ± 0.9	9.9 ± 1.0
*R* (nm)	5.1 ± 0.6	6.0 ± 0.7	7.5 ± 0.9	8.1 ± 1.0
i-TS calculation (nm)	6.4	7.0	6.9	7.6
