# Supplementary material for: Tracing temperature in a nanometer size region in a picosecond time period
Source: Sci Rep. 2015 Aug 21;5:13363. doi: 10.1038/srep13363 (PMC4543984; doi:10.1038/srep13363)
Supplement: Supplementary Information [file srep13363-s1.doc]

**Supplementary information**

**“Tracing temperature in a nanometer size region in a picosecond time period”**

*by Kaoru Nakajima, Takumi Kitayama, Hiroaki Hayashi, Makoto Matsuda, Masao Sataka, Masahiko Tsujimoto, Marcel Toulemonde, Serge Bouffard, and Kenji Kimura*

**1. Estimation of electron-phonon mean fee path**

The evolution of the temperature distribution around the impact position can be calculated using the inelastic thermal spike (i-TS) model. In the i-TS model, the heat diffusion in time *t* and space *r* (radial distance from the ion path) is described by the following differential equations [1],

$C_{e}\left(T_{e}\right)\frac{\partial T_{e}}{\partial t}=\frac{1}{r}\frac{\partial}{\partial r}\left[rK_{e}\left(T_{e}\right)\frac{\partial T_{e}}{\partial r}\right]-g\left(T_{e}-T_{a}\right)+A(r,v,t)$, (1)

$C_{a}\left(T_{a}\right)\frac{\partial T_{a}}{\partial t}=\frac{1}{r}\frac{\partial}{\partial r}\left[rK_{a}\left(T_{a}\right)\frac{\partial T_{a}}{\partial r}\right]+g\left(T_{e}-T_{a}\right)$, (2)

where *T_e, a_, C_e, a_* and *K_e, a_* are the respective temperature, specific heat and thermal conductivity of the electronic and atomic subsystems, *g* is the electron-phonon coupling parameter, *A*(*r*, *v*, *t*) is energy input into the electronic subsystem from the electronic energy loss, and *v* is the ion velocity. The energy lost by the projectile ion is sheared between electrons and then gradually transferred to atoms by the electron-phonon interaction. The parameter *g* is linked to the electron-phonon mean free path *λ* by the relation *λ*^2^ = *K_e_/g.* For crystalline Si_3_N_4_, *λ* can be estimated to be 4.3 nm from the empirical relation between *λ* and band gap energy (5.3 eV for Si_3_N_4_) [2]. Because there is no data available for a-SiN, we estimated *λ* so that the observed track radius can be reproduced by the i-TS calculation. The observed ion tracks in a-SiN consist of a density-reduced core surrounded by a density enhanced shell. Formation of such a core-shell structure is understood by the following scenario. When the temperature surpasses the boiling temperature due to the local heating caused by the ion impact the boiled region pushes the surrounding region. This results in density reduced core region. Because of the large compressibility of melt the surrounding melted region are compressed. Thus the core-shell structure is produced and the core (shell)

**Figure S1** Core and shell radii of ion tracks produced by swift heavy ions in a-SiN. The symbols show the observed results. The lines show the results of the i-TS calculations with *λ* = 2, 3 and 4 nm. The calculated result with *λ* = 3 nm agrees with the observed results.

region corresponds to the region where temperature surpassed the boiling (melting) temperature. This core-shell structure is quenched due to rapid heat diffusion and the structure remains. The i-TS model in combination with this scenario (called two threshold model) can reproduce the observed core and shell radii of the ion tracks produced in various materials, such as a-SiO_2_, CaF_2_ and so on [3, 4]. We employed the two threshold model and calculated the core and shell radii of the ion tracks produced in a-SiN with various *λ*. Figure S1 shows the comparison between the calculated radii with the observed results [5, 6]. The calculated result with *λ* = 3 nm agrees with the observed one very well. The obtained value, *λ* = 3 nm, is consistent with the fact that *λ* for amorphous materials is significantly lower than that for the same material in its crystalline phase (*λ* = 4.3 nm for crystalline Si_3_N_4_).

**2. Desorption of Pt nanoparticle**

As is suggested in the end of the paper, more detailed temperature distribution may be deduced using nanoparticles of other materials. Here, the result of platinum nanoparticles is briefly shown. A small amount of platinum was vapor deposited on a-SiN films at room temperature under a vacuum of 10^-4^ Pa. The Pt-deposited a-SiN film was irradiated with 420 MeV Au ions on the rear surface. Figure S2 shows an example of the observed TEM bright field images of the irradiated sample. The Pt nanoparticles are seen as dark spots and the ion tracks are seen as bright spots. It is clearly seen that the platinum nanoparticles disappeared from the vicinity of the ion track. By analyzing the observed TEM images in the same way described in the paper, the radius of the Pt-nanoparticle-cleared region was estimated to be 5.6 ± 0.6 nm. This is smaller than the observed radius for Au nanoparticles (8.1 ± 1.0 nm) in accordance with the fact that the melting temperature of Pt is higher than Au. The result is compared with the i-TS calculation together with the result of


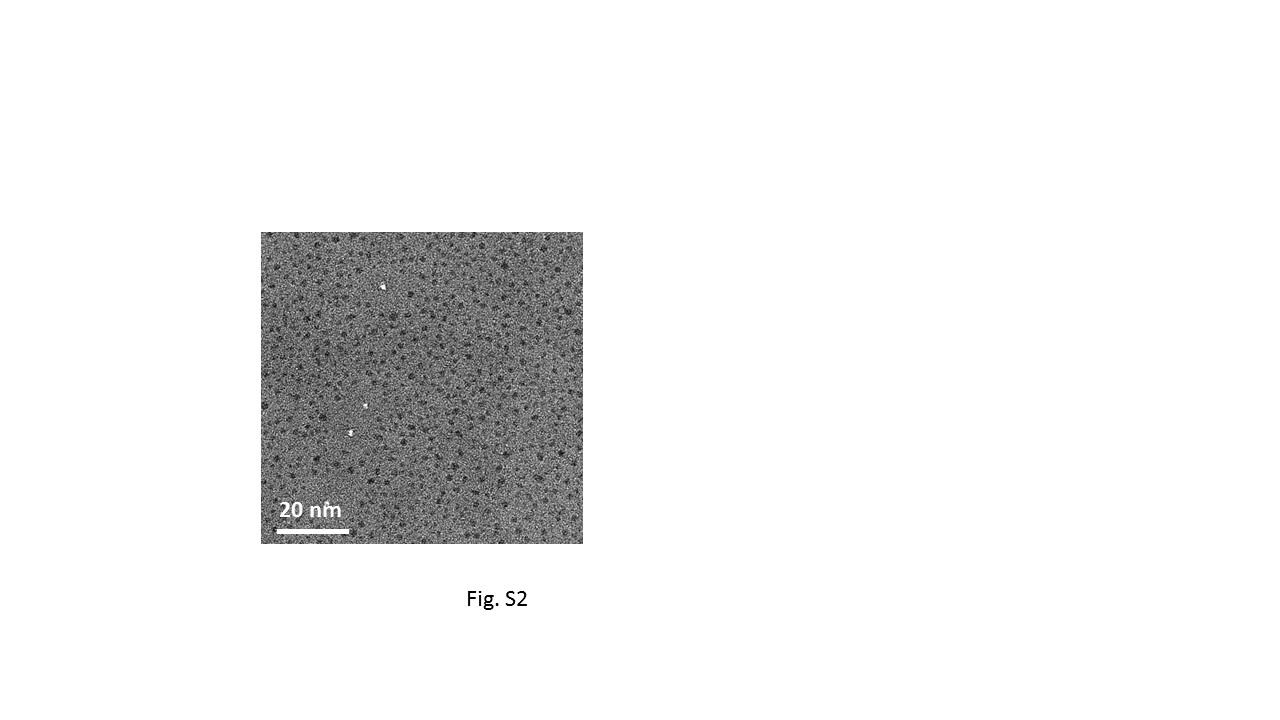


**Figure S2** TEM bright field image of platinum-deposited a-SiN film irradiated with 420 MeV Au ions on the rear surface. The ion tracks are seen as bright spots. The platinum nanoparticles disappeared from the vicinity of the ion track.

Au nanoparticles in Fig. S3. Both observed results agree with the i-TS calculation, indicating that the proposed method of temperature tracing works well.

**Figure S3** The temperature of local heating upon impact of 420 MeV Au ions on a-SiN estimated from the observation of the nanoparticle desorption (circles). The result of the i-TS calculation is shown by a solid line.

**References**

1. Toulemonde, M., *et al*. Experimental phenomena and thermal spike model description of ion tracks in amorphisable inorganic insulators. Mat. Fys. Medd. **52**, 263-292 (2006).

2. Toulemonde, M., Assmann, W., Dufour, C., Meftah, A. & Trautmann, C., Nanometric transformation of the matter by short and intense electronic excitation: Experimental data versus inelastic thermal spike model. Nucl. Instrum. Methods Phys. Res., Sect. B **277**, 28-39 (2012).

3. Kluth, P. *et al.* Fine structure in swift heavy ion tracks in amorphous SiO_2_. Phys. Rev. Lett. **101**, 175503 (2008).

4. Toulemonde, M. *et al*, Dense and nanometric electronic excitations induced by swift heavy ions in an ionic CaF_2_ crystal: Evidence for two thresholds of damage creation. Phys. Rev. B **85**, 054112 (2012).

5. Canut, B. *et al*, Ion-induced tracks in amorphous Si_3_N_4_ films. Nucl. Instrum. Methods Phys. Res., Sect. B **266**, 2819-2823 (2008).

6. Kitayama, T. *et al,* Formation of ion tracks in amorphous silicon nitride films with MeV C_60_ ions. Nucl. Instrum. Methods Phys. Res., Sect. B **356**, 22-27 (2015).
